# Supplementary material for: Regulation of B-Cell Receptor Signaling and Its Therapeutic Relevance in Aggressive B-Cell Lymphomas
Source: Cancers (Basel). 2022 Feb 9;14(4):860. doi: 10.3390/cancers14040860 (PMC8870007; doi:10.3390/cancers14040860)
Supplement: Supplementary file 1 [file cancers-14-00860-s001.zip › cancers-1556992-supplementary.pdf]

Supplemental Table S1. Recruiting information and preliminary results of clinical trials with targeted-BCR inhibition as single agent or combinatorial treatments

Targets	Drug / Regimen	Clinical Trial	Phase	Nb pts	Status	Conditions	Response Data	Reference
**BTK**	Abivertinib	NCT03060850	1	184	Unknown status	B-cell Lymphomas	PR 61.5% AEs Grade 3/4 47.6%	[181]
**BTK**	BGB-16673	NCT05006716	1	76	Recruiting	B-cell Lymphomas	NA	NA
**BTK**	CT-1530	NCT02981745	1 & 2	200	Recruiting	R/R nHL, R/R CLL, MCL, DLBCL	NA	NA
**BTK**	HZ-A-018	NCT04173455	1	46	Recruiting	B-Cell Lymphomas	NA	NA
**BTK**	ARQ-531	NCT03162536	1 & 2	190	Recruiting	No	No drug related AEs	[182]
**BTK**	NX-2127	NCT04830137	1	130	Recruiting	R/R B-cell Lymphomas	NA	NA
**BTK**	Orelabrutinib	NCT04438005	2	85	Recruiting	DLBCL	NA	NA
**BTK**	Pirtobrutinib	NCT04662255	3	500	Recruiting	MCL	NA	NA
**BTK**	Zanubrutinib	NCT05068440	2	66	Not yet recruiting	R/R DLBCL	NA	NA
**PI3K**	YY-20394	NCT03757000	1	42	Unknown status	B-cell Lymphomas	ORR 64% PFS 255 days	[183]
**BTK**	Acalabrutinib + CAR-T cell	NCT04257578	1 & 2	40	Recruiting	DLBCL, Transformed FL	NA	NA
**BTK**	Acalabrutinib + Chemotherapy	NCT03736616	2	47	Recruiting	DLBCL	NA	[184]
**BTK MDM2**	Acalabrutinib + KRT-232	NCT04502394	1 & 2	84	Recruiting	DLBCL, CLL	NA	[185]
**BTK**	Acalabrutinib + R-CHOP	NCT04978584	2	60	Not yet recruiting	DLBCL	NA	NA
**BTK**	Acalabrutinib + R-CHOP	NCT04546620	2	453	Not yet recruiting	DLBCL	NA	NA
**BTK**	Acalabrutinib + Immuno-chemotherapy	NCT04002947	2	132	Recruiting	DLBCL	NA	NA
**BTK TLR9**	Ibrutinib + SD-101 + Radiation	NCT02927964	1 & 2	30	Recruiting	FL, MZL, MCL	ORR 50%	[186]
**BTK**	Ibrutinib + CAR-T cell	NCT04234061	2	20	Recruiting	Recurrent MCL	NA	[187]
**BTK**	Ibrutinib + Chemotherapy	NCT02443077	3	302	Recruiting	R/R ABC DLBCL	NA	[188]
**BTK ROR1**	Ibrutinib + Cirmtuzumab	NCT03088878	1 & 2	160	Recruiting	CLL, SLL, MCL	ORR 83% CR 50%	[189]
**BTK**	Ibrutinib + EPOCH-R	NCT03220022	1	54	Recruiting	DLBCL	NA	NA
**BTK CD20**	Ibrutinib + Obinutuzumab	NCT03198026	2	30	Recruiting	B-cell Lymphomas	NA	NA
**BTK**	Ibrutinib + Pevonedistat	NCT03479268	1	30	Recruiting	R/R CLL, R/R DLBCL, R/R FL, R/R MCL, R/R MZL	NA	NA
**BTK CD20**	Ibrutinib + Rituximab + Bendamustine	NCT02747732	2	72	Recruiting	R/R DLBCL	NA	NA

**BTK MIL63**	Orelabrutinib + Ab MIL62	NCT04304040	1 & 2	120	Recruiting	R/R B-cell Lymphomas	ORR 70%	[190]
**BTK**	Pirtobrutinib + Rituximab + Venetoclax	NCT03740529	1 & 2	860	Recruiting	CLL, WM, MCL, MZL, SLL	ORR 86% AEs in less than 10%	[191]
**BTK**	Zanubrutinib + R-CHOP	NCT04835870	2	20	Recruiting	ABC DLBCL	NA	NA
**BTK CD20**	Zanubrutinib + Rituximab + Lenalidomide	NCT04436107	1	67	Recruiting	R/R DLBCL	NA	NA
**BTK mTOR**	DTRMWXHS-12 + Everolimus + Pomalidomide	NCT04305444	2	120	Recruiting	RR B-cell Lymphomas	NA	NA
**BTK PI3K PD1**	Zanubrutinib + BGB-10188 + Tislelizumab	NCT04282018	1 & 2	150	Recruiting	CLL, FL, MZL, MCL, DLBCL	NA	NA
**BTK PI3K**	TG1701 + YY-20394	NCT04948788	1 & 2	102	Recruiting	R/R B-cell Lymphomas	NA	NA
**BTK CD20 PI3K**	TG1701 + Ublituximab + Umbralisib	NCT03671590	1	200	Recruiting	B-cell Lymphomas	ORR 82.3% CR 23.5%	[192]
**PI3K**	Copanlisib + R-EPOCH	NCT04933617	1	34	Not yet recruiting	BL, DLBCL, GCB DLBCL	NA	NA
**PI3K**	Copanlisib + Immuno-chemotherapy	NCT04156828	1	21	Recruiting	FL, DLBCL	NA	NA
**PI3K**	Duvelisib + CAR-T cell	NCT04890236	Early 1	20	Not yet recruiting	R/R DLBCL	NA	NA
**PI3K PD1**	Umbralisib + Pembrolizumab	NCT03283137	1	36	Recruiting	B-cell Lymphomas	NA	NA
**PI3K CD20**	Umbralisib + Ublituximab + Lenalidomide	NCT04635683	1	42	Recruiting	R/R MZL, R/R FL, R/R MCL, R/R WM	NA	NA
